# A Registry for Evaluation of Efficiency and Safety of Surgical Treatment of Cartilage Defects: The German Cartilage Registry (KnorpelRegister DGOU)

**DOI:** 10.2196/resprot.5895

**Published:** 2016-06-29

**Authors:** Julia Maurer, Birgit Grotejohann, Carolin Jenkner, Carla Schneider, Thomas Flury, Adrian Tassoni, Peter Angele, Jürgen Fritz, Dirk Albrecht, Philipp Niemeyer

**Affiliations:** ^1^ Clinical Trials Unit Medical Center - University of Freiburg Faculty of Medicine, University of Freiburg Freiburg im Breisgau Germany; ^2^ Department Trauma Surgery University Hospital Regensburg Regensburg Germany; ^3^ Sporthopaedicum, Straubing Berlin, Regensburg, München Germany; ^4^ Orthopädisch Chirurgisches Centrum Tübingen Tübingen Germany; ^5^ Klinik im Kronprinzenbau Reutlingen Reutlingen Germany; ^6^ Department of Orthopedics and Trauma Surgery Medical Center - University of Freiburg Faculty of Medicine, University of Freiburg Freiburg im Breisgau Germany

**Keywords:** ankle joint, cartilage defect, chondral defect, hip joint, knee joint, patient registry

## Abstract

**Background:**

The need for documentation in cartilage defects is as obvious as in other medical specialties. Cartilage defects can cause significant pain, and lead to reduced quality of life and loss of function of the affected joint. The risk of developing osteoarthritis is high. Therefore, the socioeconomic burden of cartilage defects should not be underestimated.

**Objective:**

The objective of our study was to implement and maintain a registry of all patients undergoing surgical treatment of cartilage defects.

**Methods:**

We designed this multicenter registry for adults whose cartilage defects of a knee, ankle, or hip joint are treated surgically. The registry consists of two parts: one for the physician and one for the patient. Data for both parts will be gathered at baseline and at 6-, 12-, 24-, 36-, 60-, and 120-month follow-ups.

**Results:**

To date, a wide range of German, Swiss, and Austrian trial sites are taking part in the German Cartilage Registry, soon to be followed by further sites. More than 2124 (as of January 31, 2016) cases are already documented and the first publications have been released.

**Conclusions:**

The German Cartilage Registry addresses fundamental issues regarding the current medical care situation of patients with cartilage defects of knee, ankle, and hip joints. In addition, the registry will help to identify various procedure-specific complications, along with putative advantages and disadvantages of different chondrocyte products. It provides an expanding large-scale, unselected, standardized database for cost and care research for further retrospective studies.

**Trial Registration:**

German Clinical Trials Register: DRKS00005617; https://drks-neu.uniklinik-freiburg.de/ drks_web/navigate.do?navigationId=trial.HTML&TRIAL_ID=DRKS00005617 (Archived by WebCite at http://www.webcitation.org/6hbFqSws0)

## Introduction

Isolated cartilage defects are common orthopedic disorders in middle-aged patients that are typically associated with pain, reduced quality of life, and loss of function of the affected joint [1,2]. In fact, chondral defects have been described in 34% to 62% of knee arthroscopies [3-6]. They tend to progress to osteoarthritis, as spontaneous healing is rare, and can therefore be considered a potential risk factor or precondition for joint degeneration [7].

As of 2008, nearly 27 million US adults aged 25 and older have clinical osteoarthritis [8]. Osteoarthritis is the fourth most frequent cause of hospital admission in the United States and the leading cause of joint replacement surgery [9]. In 2009, in the United States 905,000 knee or hip replacements were conducted, resulting in treatment costs of US $42.3 billion [9]. In sum, osteoarthritis is one of the major causes of global disability and is a socioeconomic burden that will most likely soon become a substantial problem for global health systems [10,11]. Therefore, it is very important to cure cartilage defects in the first place.

Concerning cartilage repair techniques, several therapies have been established, which can be divided into two major groups: bone marrow stimulation techniques and transplantation techniques [12,13]. Despite the fact that the number of randomized controlled trials (RCTs) on cartilage repair has increased significantly over the years, RCTs aim only at direct comparison between two surgical procedures, such as the comparison between arthroscopic microfracturing and autologous chondrocyte implantation [14-18]. In addition, only a highly selected patient population is considered in these trials. Real-life clinical data are hardly ever considered.

The group of Engen et al [11] published a study on this issue and came to the final conclusion that only approximately 4.5% of patients with cartilage defects are represented by RCTs. Jakobsen et al [19] stated that promising results of cartilage repair studies have to be interpreted carefully due to their low methodological quality. Against this background and based on the fact that some scientific questions, such as a detailed analysis of surgical complication, and the influence of sex, overweight, and other factors, cannot be investigated in RCTs, many experts think that RCTs should be supplemented by well-designed observational studies [20-24]. Thus, we have initiated this multicenter patient registry to 1) systematically describe the current medical care situation of patients undergoing surgical treatment of their cartilage defect, 2) compare competing cartilage therapies regarding their outcomes, procedure-specific complication rates, and symptom relief by collecting real-life clinical data, 3) identify putative advantages and disadvantages of various chondrocyte products in daily clinical care, 4) develop new hypotheses on cartilage repair techniques as a basis for future RCTs and to test outcomes of former RCTs in a larger and more representative population, and 5) evaluate the efficiency and safety of surgically treated cartilage defects of knee, hip, and ankle joints, independent of strict patient characteristics or surgical procedure.

Here we describe the study design and layout of the German Cartilage Registry, which is to our knowledge the first patient registry for this indication worldwide.

## Methods

### Study Design

The German Cartilage Registry is an observational and international multicenter registry that was initiated by the *Arbeitsgemeinschaft Klinische Geweberegeneration* (Working Group Clinical Tissue Regeneration) of the German Society for Orthopaedics and Trauma (DGOU) in 2013. It is a purely scientifically motivated project and as a consequence independent of the interests of industrial partners. The study is conducted in accordance with the Declaration of Helsinki and registered at germanctr.de (DRKS00005617).

The registry investigates the efficiency and safety of surgical treatment of cartilage defects in patients under real-life conditions. In October 2013, the assessment started with the documentation of cartilage defects of the knee. The modules for cartilage defects of the ankle and hip joint were implemented 1 year later.

**Ethics Approval**

Depending on individual state’s law, investigators consult the responsible ethics committee before starting the study at their site. At their request, investigators are supported by the Clinical Trials Unit (CTU; Medical Center - University of Freiburg, Freiburg, Germany) in preparing the essential documents for submission (first approval in Freiburg on March 13, 2013, internal number 105/13). So far, 33 ethics committees have welcomed the implementation of the German Cartilage Registry in their jurisdiction.

After consulting the ethics committee, investigators are allowed to take part in the German Cartilage Registry.

### Study Population

All patients aged ≥18 years who meet the following criteria are eligible to take part in the German Cartilage Registry: 1) they have had surgical treatment of cartilage defects of a knee, ankle, or hip joint at a participating site, 2) they have given written informed consent, 3) they have a personal email address.

### Procedure and Data Collection

Only after the patient has signed the written informed consent the investigator is allowed to register the patient in the database. We recommend that this registration procedure takes place immediately after the surgery is completed. Thus, the investigator can type in the following mandatory baseline data at the same time: initially, the date of surgery, the patient’s identification, and the patient’s email address to generate a new case; and subsequently, basic information concerning patient history and treatment technique. In the course of 6-, 12-, 24-, 36-, 60-, and 120-month follow-ups, the physician can document further optional data.

**Figure 1:**
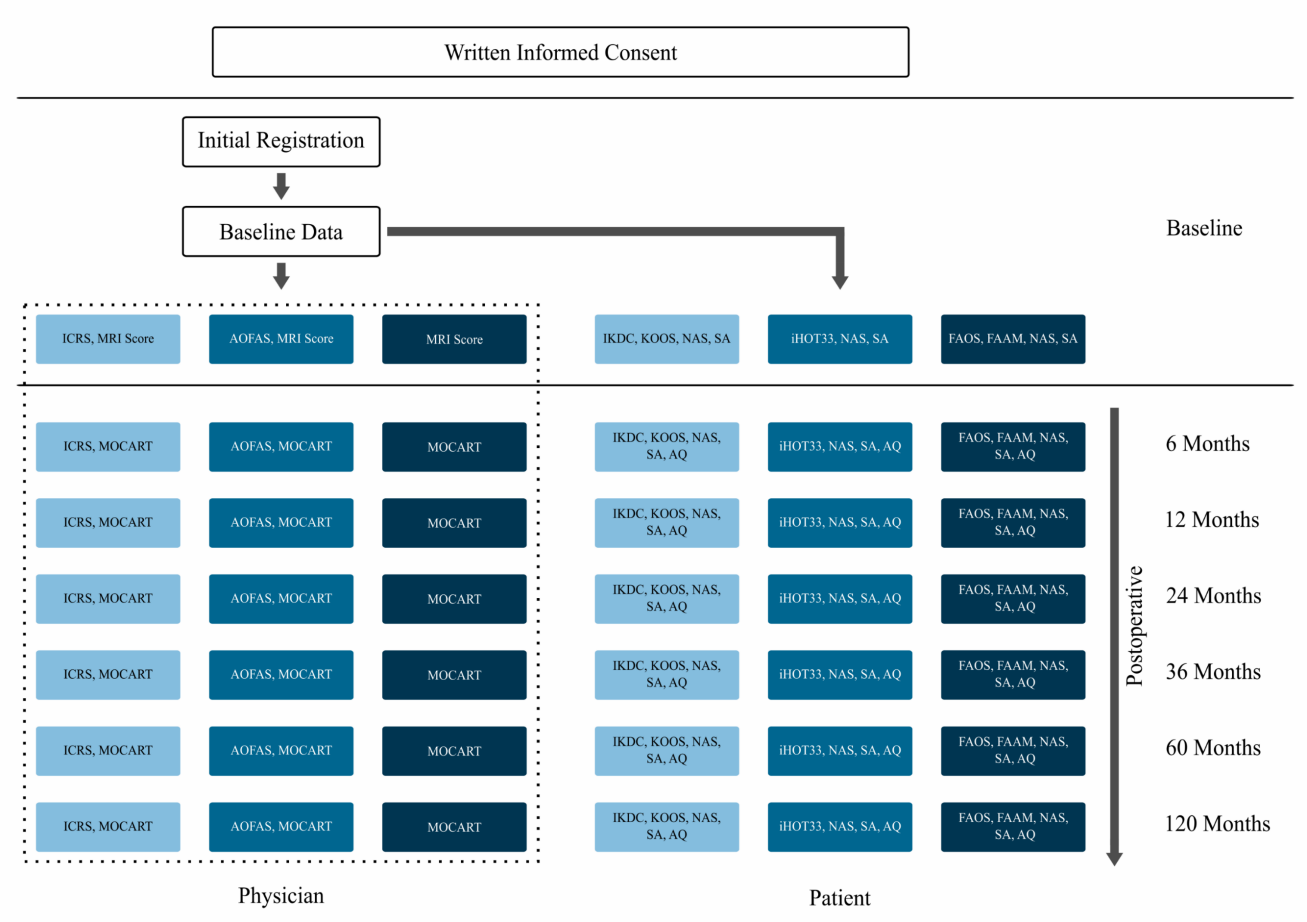
Flow chart of the German Cartilage Registry and questionnaires deployed to physicians and patients. AOFAS: American Orthopaedic Foot & Ankle Society; AQ: additional questions; FAAM: Foot and Ankle Ability Measure; FAOS: Foot and Ankle Outcome Score; ICRS: International Cartilage Repair Society; iHOT33: International Hip Outcome Tool-33; IKDC: International Knee Documentation Committee; KOOS: Knee injury and Osteoarthritis Outcome Score; MOCART: magnetic resonance observation of cartilage repair tissue; MRI: magnetic resonance imaging; NAS: numeric analog scale for pain description; SA: sports activities.

The day following initial data entry by the physician, the patient automatically receives an email inviting him or her to fill in a questionnaire for baseline data. Additionally, the patient receives an invitational email at 6-, 12-, 24-, 36-, 60-, and 120-month follow-ups to complete the questionnaire. If the patient does not complete the form within a given time limit, an email reminder is sent automatically. If the patient still does not fill in the questionnaire, the trial site seeks personal contact. Figure 1 shows the flow chart of the German Cartilage Registry in detail, naming all deployed questionnaires. Light blue represents all questionnaires that are used in the knee part, medium blue shows the questionnaires deployed in the hip part, and dark blue displays all questionnaires used in the ankle part of the German Cartilage Registry. Completion of the questionnaires shown in the dotted box is optional. Answering all other questionnaires is mandatory.

**Instruments**

The German Cartilage Registry consists of two parts: one for the physician and one for the patient. At baseline, the physician section includes mandatory information on patient-specific characteristics (age, sex, smoking behavior, weight and height, as well as varus or valgus malalignment), the preliminary operation(s), all surgical procedures performed on the injured joint (including defect-specific characteristics), and therapy characteristics.

Furthermore, the physician can fill in a premagnetic resonance imaging score (similar to the magnetic resonance observation of cartilage repair tissue, or MOCART, score), as well as joint-specific scores, such as the International Cartilage Repair Society (ICRS) score (equal to International Knee Documentation Committee, IKDC, objective score) for the knee joint and American Orthopaedic Foot & Ankle Society (AOFAS) for the ankle joint [25,26]. Investigator’s data entry at 6-, 12-, 24-, 36-, 60-, and 120-month follow-ups is optional. But there is the opportunity to document joint-specific scores, such as MOCART [27,28], AOFAS, and ICRS (see Figure 1).

At all times, the patient’s questionnaire consists of a numeric analog scale for pain description, a few questions about sports activities, and joint-specific, validated, and standardized instruments, such as IKDC subjective score and Knee injury and Osteoarthritis Outcome Score (for the knee part) [29-33], International Hip Outcome Tool-33 (hip part) [34,35], Foot and Ankle Outcome Score, and Foot and Ankle Ability Measure (ankle part) [36-38]. At 6-, 12-, 24-, 36-, 60-, and 120-month follow-ups, 3 additional questions ask about the patient’s satisfaction with the surgical treatment at baseline and further surgeries (see Figure 1).

### Data Entry

The Web-based remote data entry system called RDE-LIGHT was developed by the CTU of the Medical Center - University of Freiburg as an electronic data entry interface and data management system for clinical studies and other projects in clinical research. Data are collected paperless and directly on site via an Internet browser. The RDE-LIGHT system displays the questionnaires in a structured view in the main window, indicating the status of the questionnaires as traffic light colors. Questionnaires are based on HTML. RDE-LIGHT is available in various languages and validated according to GAMP 5 (ISPE, Tampa, FL, USA). Furthermore, it fulfills all requirements of good clinical practice.

The RDE-LIGHT system applies established security standards such as cryptographic security protocols (secure socket layer/transport layer security), user authentication protocols, and authorization concepts. For example, investigators can access data only of their own site, while the system denies unauthorized access. Data transfer to the database is encrypted and secured. The server is located in the Medical Computer Department of the Medical Center - University of Freiburg, with strict access control. Hence, common concepts of data protection are implemented. Changes to the database and the underlying system are logged, saved, and archived regularly to ensure end-to-end tracking.

**Figure 2:**
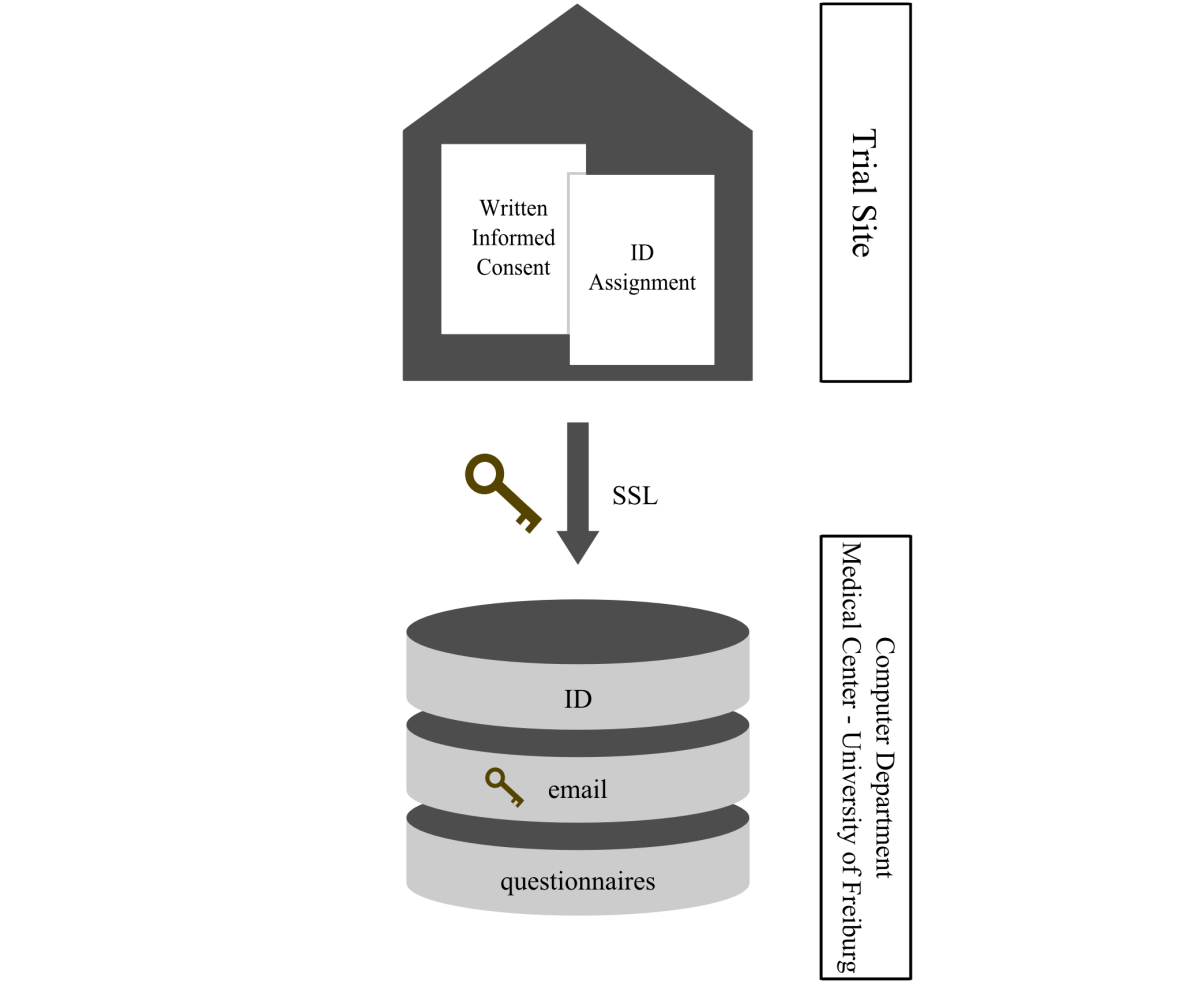
Data storage location for the German Cartilage Registry. ID: identification; SSL: secure socket layer.

When working with personal data, the CTU encourages involved researchers to use pseudonyms to prohibit the identification of their patients. The patients’ names and contact details (email address) will be kept confidential and are available to the research team only for contact purposes. Any data presented publically will ensure participants' anonymity. In order to be able to automatically send emails to the patients when new questionnaires have to be completed, it is necessary to access the patients’ email addresses in the system’s database. As the email address is part of the patients’ personal information, it is stored in an encrypted way in the database using password-based encryption with MD5 and Triple Data Encryption Standard. Figure 2 illustrates the data storage location and clearly shows that the email addresses are separated from the physicians’ and patients’ questionnaires, as well as the patients’ identification, in a well-protected way.

### Statistical Analysis

After approval from the *Arbeitsgemeinschaft Klinische Geweberegeneration* (Working Group Clinical Tissue Regeneration), every participating physician is allowed to publish the full set of anonymized data available at that time. Data will always be prepared by an experienced biostatistician of the CTU. We are planning several descriptive analyses concerning the structure and composition of the registry. Analyses will be done by first specifying several different research questions (eg, efficacy of certain therapies in real-life datasets) and prespecifying inclusion criteria for these specific questions. Independently, every investigator is allowed to download his or her own data set for anonymized statistical evaluations. However, it is important to keep in mind that registry data need special care in the analysis, as populations are unbalanced and several sources of bias can be present, such as in confounding variables. Therefore, the results must be interpreted very carefully.

### Quality Assurance

The registry is being implemented and maintained by the CTU of the Medical Center- University of Freiburg. The CTU is member of the network of coordinating centers for clinical trials in Germany [39] and offers profound expertise in all areas of clinical trial planning, conduct, and analysis, in both universities and industry. The CTU is involved in about 250 trials a year.

Skilled and experienced staff of the CTU offer email and telephone support for any emerging problems. Additionally, documents, user manuals, and Web-based instructions via video may help to assist physicians and other personnel at the site. A query management system helps to identify patients and physicians who did not fill in the mandatory questionnaires.

## Results

**Figure 3:**
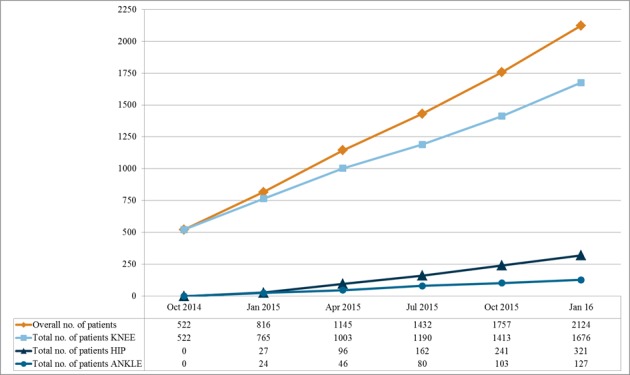
Total number of patients in the German Cartilage Registry as of January 31, 2016.

At time of data collection for this paper, 100 German trial sites and 5 trial sites in Austria and Switzerland are taking part in the German Cartilage Registry. Among these are university medical centers and private hospitals, doctors’ surgeries, and outpatient surgical centers. As of January 31, 2016, a total of 2124 patients have been registered (see Figure 3) and the first clinical results have been published [40-42].

## Discussion

The primary aim of this multicenter registry is to assess the efficiency and safety of surgically treated cartilage defects of knee, hip, and ankle joints and to subsequently provide future patients with their best treatment option. Therefore, we are collecting as much valuable information as possible on a preferably heterogeneous group of people who have been treated in day-to-day clinical practice.

In the following section we highlight the strengths of the German Cartilage Registry and discuss the known limitations to this project.

### Complementing Data from RCTs

In recent years, there has been a focus on RCTs in cartilage repair, since they still are the highest level of clinical research [43]. Nevertheless, due to strict inclusion and exclusion criteria, study populations in many RCTs do not completely represent clinical routine and the entire population of patients with cartilage defects. In fact, the number of patients who are eligible for RCTs in the field of cartilage defects is estimated to be only around 4.5% [11]. Hence, the vast majority of patients are not represented by RCTs, since they do not qualify for different reasons, such as an increased body mass index or concomitant pathologies. In addition, important patient-related factors such as smoking and being overweight have been proven to significantly influence the outcome of cartilage repair techniques, but they cannot be analyzed by RCTs for methodological reasons [44-46]. This also applies to pathology-related parameters such as the influence of defect size or detailed defect location [46]. Furthermore, concomitant pathologies are considered to be exclusion criteria in most RCTs but are frequently present in cartilage repair patients. All of these factors underline the necessity of not exclusively relying on findings of RCTs, but to complement the results of RCTs with data from well-designed observational studies (eg, registries), and, therefore, to reassess findings of RCTs in daily clinical use.

### Selection Bias

Due to organizational or other limitations, we cannot guarantee that every single patient with a surgically treated chondral defect of a knee, hip, or ankle joint will be documented in the system. For instance, for small- or medium-sized medical health providers, the additional workload for data input may seem too high. But we tried to keep the administrative effort as small as possible by allowing the physicians to register a patient immediately after surgery has been completed, although the first patient questionnaire refers to the complaints before surgery. In this way, the physician can record all mandatory data at once. Furthermore, we tried to include as many patient characteristics as possible that are thought to affect outcomes.

### Data Quality

No onsite clinical monitoring is provided to assure the quality of entered data, and the respective sites are solely responsible for data input. Nevertheless, quality parameters need to be established and carefully applied. For instance, we have to observe the follow-up rate, which is crucial in any type of clinical research. Therefore, a validation study of recorded data will have to follow.

### Expansion of the Registry

Additional sites in German-speaking countries, namely Austria and Switzerland, have already been affiliated and others will be approached to join the registry.

Further information is available on the KnorpelRegister website [47].

## Abbreviations

AOFAS: American Orthopaedic Foot & Ankle Society
CTU: Clinical Trials Unit
DGOU: German Society for Orthopaedics and Trauma
ICRS: International Cartilage Repair Society
IKDC: International Knee Documentation Committee
MOCART: magnetic resonance observation of cartilage repair tissue
RCT: randomized controlled trial
